# Nephritis in Summer

**Published:** 1903-07

**Authors:** William F. Waugh

**Affiliations:** Chicago


For Texas Medical Journal.

### Nephritis in Summer.

BY WILLIAM F. WAUGH, M. D., CHICAGO.

Patients with any form of Bright's disease need careful watching in summer. The heat and the free use of water are apt to occasion such free sweating that the excretion of urine falls too low. And

Bouchard's observation was that a pint of urine eliminated as much as ten pints of perspiration.

The diet should be very carefully limited, avoiding meats as much as possible. Juicy fruits and berries should form the bulk of the food. Use only hot beverages. Eschew spices and water-cress. Alcohol is always injurious, but never so much as in the dog days. Buttermilk is as nearly an ideal food as can be imagined. Let it be taken pleasantly cool, not iced, and drank or rather sipped slowly.

I believe in keeping woolen underclothes on nephritics all the year around, and the thinnest grades are not uncomfortable in July. This applies also to the stockings.

The urine should be tested very often, for total excretion of solids as well as for albumin. Any decrease in excretion must be promptly met, as the heat increases the tendency to uremia, and this renders the patient especially liable to sunstroke.

In desquamative nephritis I am more and more convinced of the value of arbutin, given persistently in doses of one-sixth gr. before and after each meal and on going to bed, and continued for a year or more. With a suitable diet and proper hygiene, the malady slowly subsides, until, in many cases, possibly in most, all evidences of the disease disappear. I might except one evidence from this—the tendency to headache on exposure to cold or wind shows a permanent loss of the reserve renal power. The patient has recovered with a loss of a portion of the kidney structure. This, I believe, is the rule in such cases.

In cirrhotic nephritis the conditions are totally different. The diet is about the same, since in both forms the indication is that of giving the suffering organs as much rest, or as little work, as the necessities of life admit. We seek to reduce the work of the kidneys to the lowest possible point, by reducing the quantity of nitrogenous food to the lowest point compatible with comfortable life. Every bit of necessary nitrogenous food means an increase in the toxins that the kidneys must elminate.

But the element of greatest danger in cirrhotic nephritis lies in the increased arterial tension, and the remedy is veratrine. It seems singular that the profession has neglected this most admirably suitable remedy, while availing itself of glonoin. The latter relaxes arterial tension and gives such relief that its use is established as a routine. But the effect is evanescent; it is quickly manifested and rapidly subsides. Veratrine is slow in getting to work, but by its judicious administration the vascular tension may be maintained at the desired point for months, without harm or discomfort. It is one of the most uniform and manageable of remedies, of unsurpassed power, and singularly provided with safeguards that prevent danger. As a universal stimulant of the entire excretory apparatus it has no rival.

The mistake has been in the doses. Give gr. 1-134 and repeat every hour till the arterial tension has subsided to the normal point, and then give the remedy just often enough to maintain this circulatory equilibrium. Overdoses will manifest themselves by nausea or diarrhea.

Indications of uremia call for larger or more frequent doses, commensurate with the danger. In emergencies veratrine may be administered hypodermically. It is best given diluted, as even in small doses it may irritate a very sensitive stomach.

